# Fungal endocarditis with heart valve replacement and atrial fibrillation posing a treatment challenge: A case report

**DOI:** 10.1097/MD.0000000000022487

**Published:** 2020-11-25

**Authors:** Xiaoxia Zhu, Shugang Cao, Mingwu Xia, Chandong Ding, Rongfeng Wang

**Affiliations:** a Department of Cardiology, The Second Affiliated Hospital of Anhui Medical University; b Department of Neurology, The Affiliated Hefei Hospital of Anhui Medical University, Hefei, Anhui, P.R. China.

**Keywords:** antifungal treatment, atrial fibrillation, cerebral embolism, fungal endocarditis, heart valve replacement, warfarin

## Abstract

**Rationale:**

Fungal endocarditis (FE) is a rare disease, in which antifungal treatment is necessary. When FE is complicated with prosthetic heart valve and/or atrial fibrillation, the coadministration of antifungal agents and warfarin is inevitable. We report a case of rheumatic heart disease with atrial fibrillation who developed FE following prosthetic heart valve replacement. The international normalized ratio (INR) increased significantly during the antifungal treatment with fluconazole. A discussion of the antifungal strategy in FE patients with prosthetic heart valves and/or atrial fibrillation and the interaction between antifungal agents and warfarin was performed.

**Patient concerns:**

A 54-year-old Chinese woman experienced intermittent fevers, aphemia, and weakness in her right extremities. Her temperature was 38.7°C, and there was atrial fibrillation with heart rate 110 times/min. Neurological examination revealed that she had drowsiness, Broca aphasia, right central facial paralysis, and hemiplegia (Medical Research Council scale, upper limb grade 0, lower limb grade II).

**Diagnoses:**

Multiple infarction on magnetic resonance imaging and the occlusion of left middle cerebral artery suggested the occurrence of cerebral embolism. The presence of *Candida parapsilosis* in the results of 4 blood cultures and the existence of valve vegetation in the reexamination of echocardiogram supported the diagnosis of FE.

**Interventions:**

The patient was given antifungal therapy with fluconazol. The INR increased dramatically on the 9th day of antifungal treatment, and subcutaneous bruising occurred at the intravenous infusion site. The antagonist of vitamin K1 was used and warfarin was reduced to a smaller dosage. The antifungal agent was replaced with caspofungin.

**Outcomes:**

Her speech improved significantly, and the muscle strength of her paralyzed side reached the Medical Research Council scale of grade IV^+^. She continued to receive caspofungin for antifungal treatment with relatively stable INR and waited for heart valve surgery.

**Lessons:**

The choice of antifungal agents is often a big challenge for FE patients, especially when they need warfarin for anticoagulation. It is better to administer a low dose of warfarin while carefully monitoring the INR or choose the antifungal drugs with little or no effect on warfarin.

## 1 Introduction

Fungal endocarditis (FE) is a rare form of infective endocarditis with an extremely high mortality rate.^[1]^ An implanted prosthetic heart valve is a well-known risk factor for FE. Patients with prosthetic heart valves usually need short-term or long-term anticoagulation treatment using warfarin rather than new oral anticoagulants.^[2]^ When they are complicated with atrial fibrillation, long-term anticoagulation is necessary. Azole antifungal agents are often the first choice of empirical treatment for FE. However, azole antifungal agents usually augment the anticoagulant activity of warfarin, thereby increasing the risk of bleeding.^[3]^ It is challenging to determine whether to reduce warfarin and measure the international normalized ratio (INR) repeatedly or to replace the antifungal drugs with medications with less or no influence on warfarin. We report a case of FE manifested as cerebral embolism, in which the INR increased dramatically during antifungal treatment with fluconazole. We reversed the anticoagulant effects of warfarin with vitamin K1 and then reduced the dosage of warfarin. In the end, however, the antifungal drug was replaced with caspofungin, which does not interact with warfarin. Here, we intended to provide a concise review of the clinical features of FE, the antifungal treatment strategy for FE patients with prosthetic heart valve and/or atrial fibrillation, and the interaction between antifungal agents and warfarin.

## 2 Case presentation

A 54-year-old Chinese woman presented with intermittent fevers for half a month and aphemia and weakness in her right extremities approximately 10 hours after onset. In terms of medical history, she had been diagnosed with rheumatic heart disease complicated with mitral, tricuspid, and aortic insufficiency and atrial fibrillation for 1.5 years, and she underwent mitral and aortic bioprosthetic valve replacement and tricuspid valvuloplasty 16 months ago. She was given warfarin (3 mg/d) for secondary prevention of cerebral and systemic embolism, with an INR of 2.0 1 week before admission.

The physical examination findings were as follows: temperature (T): 38.7°C, pulse rate (P): 104 times/min, respiration (R): 20 times/min, and blood pressure: 78/56 mm Hg; there was atrial fibrillation, with heart rate 110 times/min, but no abnormal findings in the lungs and abdomen. Neurological examination revealed that she had drowsiness, Broca aphasia, right central facial paralysis, and hemiplegia (Medical Research Council scale, upper limb grade 0, lower limb grade II), while she failed to complete the sensory tests and coordinate movements. Her right Babinski sign was positive. Her initial National Institutes of Health Stroke Scale (NIHSS) score was 14.

The results of laboratory tests were as follows: coagulation tests showed that the prothrombin time was 22.30 seconds (normal range, 10.00–15.00 seconds), the activated partial thromboplastin time was 53.50 seconds (normal range, 28.00**–**45.00 seconds), and the INR was 1.98 (normal range, 0.7**–**1.6). Routine blood examination showed that the red blood cell count was 3.77 ∗ 10^12/L (normal range, 4.00**–**5.50 ∗ 10^12/L), the white blood cell count was 6.72 ∗ 10^9/L (normal range, 4.00**–**10.00 ∗ 10^9/L), the platelet count was 56.0 ∗ 10^9/L (normal range, 100.0**–**300.00 ∗ 10^9/L), and the hemoglobin was 109.0 g/L (normal range, 110.0**–**150.00 g/L). Biochemical tests demonstrated a high C-reactive protein level of 44.5 mg/L (normal range, 0**–**8.2 mg/L) but without other significant abnormal findings. Electrocardiogram revealed atrial fibrillation with a fast ventricular rate. Brain computerized topography showed no cerebral hemorrhage.

**Figure 1:**
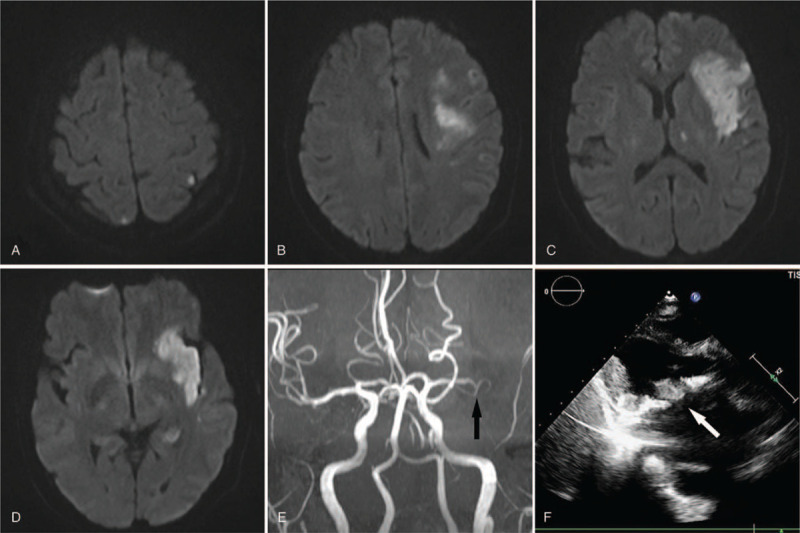
Cranial MRI. Axial diffusion-weighted imaging (DWI) showed high-signal lesions comprising the left frontal-temporal-parietal lobe, insula, hippocampus and thalamus as well as the right parietal lobe (A–D), with low apparent diffusion coefficient signals in the corresponding regions (not shown), and MR angiography (MRA) indicated the left middle cerebral artery occlusion (E, black arrows). Echocardiogram. Echocardiogram suggested the mural thrombus formation in the anterior leaflet of the mitral valve (F, white arrows). MRI = magnetic resonance imaging.

After admission, she was given anticoagulation, blood pressure stabilization, and anti-infective therapy with antibiotics and symptomatic treatment. The next day, she became alert, and her speech improved (NIHSS score: 10). Initial echocardiography showed mitral valve and aortic valve replacement with mild aortic regurgitation and left atrial enlargement. Magnetic resonance imaging revealed multiple ischemic infarcts involving the left frontal-temporal-parietal lobe, insula, hippocampus and thalamus as well as the right parietal lobe (Fig. 1A–D), and MR angiography indicated occlusion of the left middle cerebral artery (Fig. 1E). The first blood culture revealed *Candida parapsilosis* on the 5th day, but by this time, her temperature had returned to normal. The results of 3 subsequent blood culture tests were consistent with the first one, and the result of fungal D-glucan in the fourth blood culture was 207 pg/mL (normal range, <70 pg/mL). Echocardiogram reexamination suggested vegetation (1.41 cm ∗ 0.98 cm) in the anterior leaflet of the mitral valve (Fig. 1F). She was finally diagnosed with FE leading to multiple cerebral embolisms. The patient was then given antifungal therapy with fluconazole based on the drug sensitivity test. On the third day of antifungal treatment, the INR was 2.9 according to a portable coagulation detector. However, on the ninth day of antifungal treatment, subcutaneous bruising occurred at the intravenous infusion site. The INR reached 10.21, so warfarin was stopped at once. The INR decreased to 1.1 after using 10 mg vitamin K1. The next day, warfarin was reduced to a smaller dosage (2 mg/d). The replacement of the antifungal drug with caspofungin and elective surgery were suggested. However, the patient was transferred to a large hospital in Shanghai for surgical treatment. At discharge, her speech had improved significantly, and the muscle strength of her paralyzed side reached grade IV^+^ on the Medical Research Council scale. Her final NIHSS score was 3. She continued to receive caspofungin for antifungal treatment while waiting for heart valve surgery. During antifungal therapy with caspofungin, the retesting INR values fluctuated between 1.7 and 3.3 using the same dosage of warfarin (3 mg/d).

## 3 Discussion

FE, first reported by Wolfe et al^[4]^ in 1951, is a rare but fatal entity associated with high morbidity and mortality and is similar to common bacterial endocarditis in clinical manifestations.^[1]^*Candida albicans* spp. and Aspergillus spp. are the most predominant organisms causing FE. Non-*albicans* Candida spp. (eg, *C parapsilosis*, *Candida glabrata*, and *Candida tropicalis*) and other less frequent fungal species are much less common.^[1]^ Because most of the time, the blood culture is negative in over 50% of cases and it takes a long time for pathogenic fungi to grow, the early diagnosis of FE is extremely challenging but very crucial for antifungal treatment. The demonstrable valve vegetation on echocardiography is very helpful for the diagnosis.

This patient was admitted for cerebral embolism, with intermittent fever for half a month before admission. There was a predisposing factor (namely, prosthetic valve replacement) for infective endocarditis, but no evidence of infection in other areas. Moreover, the body temperature returned to normal after antibiotic treatment. Therefore, bacterial endocarditis should be initially considered, although no thrombus was found in the first echocardiography. However, unexpectedly, the results of 4 blood cultures all indicated the presence of *C parapsilosis* and the increase in fungal glucan, in support of FE. Subsequent echocardiogram reexamination also confirmed the existence of valve vegetation. The patient was finally diagnosed with FE according to the modified Duke criteria for infective endocarditis.^[5]^ The valve vegetations in FE patients are usually large, loose in texture, and easy to fall off, which will significantly increase the incidence of vascular embolism events, as in our case.

In addition, the longer course of FE, the difficulty of obtaining timely diagnosis, and more severe valve damage frame FE as a more serious condition than bacterial endocarditis and as having a poor reaction to antifungal drugs. The patient was initially treated with the antifungal agent fluconazole according to the empirical treatment and drug sensitivity test. However, she needed warfarin for anticoagulation due to the heart valve replacement and atrial fibrillation. Unfortunately, the INR increased gradually over the course of antifungal treatment, and subcutaneous bruising occurred. The inhibition of hepatic drug-metabolizing enzymes (mainly CYP2C9) is identified as the mechanism of the interaction between warfarin and azoles such as miconazole, fluconazole, voriconazole, and itraconazole, among others.^[3]^ However, such patients can only use warfarin at present, but not new oral anticoagulants. Therefore, for such patients, we could only reduce the dosage of warfarin or select antifungal drugs with little or no effect on the anticoagulant activity of warfarin, such as caspofungin. Caspofungin plays an antifungal role by inhibiting the synthesis of β(1,3)-D-glucan, a basic component of the cell walls of many yeasts and molds. The elimination of caspofungin follows a different pathway, mainly through peptide hydrolysis and/or N-acetylation, that is not involved in the elimination of warfarin.^[6,7]^ The coadministration of caspofungin and warfarin is relatively safe due to the lack of interaction between these 2 compounds.^[8]^ Moreover, caspofungin is generally well tolerated with fewer side effects than amphotericin B.^[8]^ Nevertheless, for FE patients, the effect of antifungal therapy alone is poor, so surgical treatment is also highly recommended. The combination of these 2 treatments can improve the prognosis of FE patients.^[1,9]^ Hence, the replacement of antifungal drugs and elective surgery were finally suggested for this patient.

In summary, the choice of antifungal agents is often a major challenge for FE patients when they need warfarin for anticoagulation at the same time. It is better to administer a low dosage of warfarin while carefully monitoring the INR or to choose antifungal drugs with little or no effect on warfarin on the basis of the antifungal drug sensitivity test.

## Author contributions

**Conceptualization:** Xiaoxia Zhu, Shugang Cao, Mingwu Xia, Rongfeng Wang, Chandong Ding.

**Investigation:** Xiaoxia Zhu, Shugang Cao, Rongfeng Wang.

**Methodology:** Xiaoxia Zhu, Shugang Cao, Rongfeng Wang.

**Supervision:** Rongfeng Wang, Mingwu Xia, Chandong Ding.

**Validation:** Rongfeng Wang, Chandong Ding.

**Visualization:** Xiaoxia Zhu.

**Writing – original draft:** Xiaoxia Zhu, Shugang Cao.

**Writing – review & editing:** Xiaoxia Zhu, Shugang Cao, Rongfeng Wang, Chandong Ding.
